# Superiority of the Rockwood Clinical Frailty Scale for Assessing Long‐Term Outcome in Patients Undergoing Transcatheter Aortic Valve Implantation

**DOI:** 10.1002/ccd.70380

**Published:** 2025-11-30

**Authors:** Timothy J. Bagnall, Arul Baradi, Christopher Pavitt, Huda Abu‐Own, Andrew Hill, Stanislav Hadjivassilev, Jessica Parker, Victoria Parish, Michael Michail, James Cockburn, David Hildick‐Smith, Christopher J. Broyd

**Affiliations:** ^1^ Sussex Cardiac Centre Royal Sussex County Hospital Brighton UK; ^2^ Brighton and Sussex Medical School Brighton and Hove UK

## Abstract

**Background:**

Frailty is associated with poorer short‐term outcomes after transcatheter aortic valve implantation (TAVI), and national guidelines recommend an assessment of frailty as part of the decision‐making process. However, multiple frailty indices are currently used in clinical practice, and it is unclear which has the best discrimination in TAVI patients and whether this extends into the long term.

**Aims:**

We therefore aimed to compare three commonly used frailty indices in a large TAVI cohort and examine their ability to predict 5‐year outcomes.

**Methods:**

The study population included 1059 patients from a high‐volume UK TAVI center from 2007 to 2019. Data were collected prospectively using a data set recommended by the British Cardiovascular Intervention Society (BCIS). Frailty was assessed using the Rockwood Clinical Frailty Scale (CFS), Karnofsky Performance Index, and the Katz Index of Activities of Daily Living. Follow‐up data were obtained from the NHS Spine.

**Results:**

One, three, and five‐year survival was 86.5%, 66.7%, and 44.9%, respectively. In univariate analysis, all three frailty indices were predictive of 5‐year survival (Rockwood CFS HR: 1.34 [*p* < 0.01]; Katz HR: 1.23 [*p* < 0.01]; Karnofsky HR: 1.21 [*p* < 0.01]). Three separate multivariable Cox regression models were constructed for each frailty index. In each model, the frailty indices remained predictive of 5‐year survival. ROC curve comparison, however, demonstrated the Rockwood CFS model as the best discriminator of 5‐year survival (Rockwood CFS model: AUC = 0.59, Karnofsky model: AUC = 0.55, Katz model: AUC = 0.55, *p* = 0.04); pairwise analysis further confirmed the superiority of the Rockwood CFS model.

**Conclusion:**

Our study confirms that frailty is an important predictor of long‐term outcome following TAVI. Our study suggests that of the three most commonly used scoring systems, the most useful discriminator of long‐term outcome is the Rockwood CFS.

**Abbreviations**
CFS: clinical frailty score
CVA: cerebrovascular accident
MACE: major adverse cardiovascular events
PCI: percutaneous coronary interventions
RMST: restricted mean survival time
SAVR: surgical aortic valve replacement
TAVI: transcatheter aortic valve implantation
TIA: transient ischemic attack

## 1 Introduction

Transcatheter aortic valve implantation (TAVI) is a well‐established treatment for patients with aortic stenosis and has been shown to improve survival and quality of life [1, 2]. Patient selection remains crucial in identifying patients who will have successful short and long‐term outcomes following intervention [3].

Surgical risk scores are traditionally used to select patients for surgical aortic valve replacement (SAVR), but scoring systems such as the Society of Thoracic Surgeons (STS) score and EuroSCORE have been demonstrated to be suboptimal at predicting mortality in TAVI patients [3, 4, 5, 6]. Several other clinical predictive models (CPMs) have been developed to assess peri‐procedural outcomes in patients undergoing TAVI including FRANCE‐2 [7] and ACC‐TAVI [8], but these models do not take into account frailty and are not designed to provide longer‐term insights.

Frailty is a clinical syndrome related to the ageing process through which body systems lose physiological reserves to respond to both pathological and iatrogenic stressors [9]. There is growing evidence that suggests that frailty can be associated with poorer outcomes following TAVI including death and disability in the short‐term [3, 10, 11]. Still, it is unclear if this effect is maintained in the longer term [12]. An assessment of frailty is recommended by national guidelines [6, 13] as a key part of the decision‐making process in selecting patients for TAVI and is echoed by recent evidence from the UK TAVI registry [11]. However, an assessment of frailty is often subjective and there are various multidomain frailty scales used in clinical practice. As a result of this, there can be significant variability in the assessments of frailty between different centers and countries with no clear guidance for which scale is most appropriate for TAVI patients.

This study, therefore, had two novel aims. First, to examine the effect of frailty over a long‐term follow‐up period following TAVI, and second, to establish which frailty score is most useful at predicting outcome following TAVI.

## 2 Methods

### 2.1 Patient Population

The study population included 1059 consecutive patients from a high‐volume single UK TAVI center over a 12‐year period (2007−2019). The majority of patients (96.8%) had been selected for TAVI after consideration by a multi‐disciplinary team meeting which was attended by interventional cardiologists, non‐interventional cardiologists, cardiac surgeons, radiologists, specialist nurses, and other allied health professionals. Patients were assessed face‐to‐face by at least one TAVI operator before their procedure.

### 2.2 Data Collection

Data was collected prospectively for all TAVI cases on a local bespoke database using a data set agreed by the British Cardiovascular Intervention Society. This includes information on patient demographics, risk factors, and outcome measures. All information was entered by the operator at the time of the index procedure and cross‐checked by an independent audit officer at the end of the hospital admission. Data consistency is assured by an internal audit undertaken by independent data officers within the hospitals. All data is cross‐checked before uploading to the UK Central Cardiac Audit Database (CCAD).

**Figure 1:**
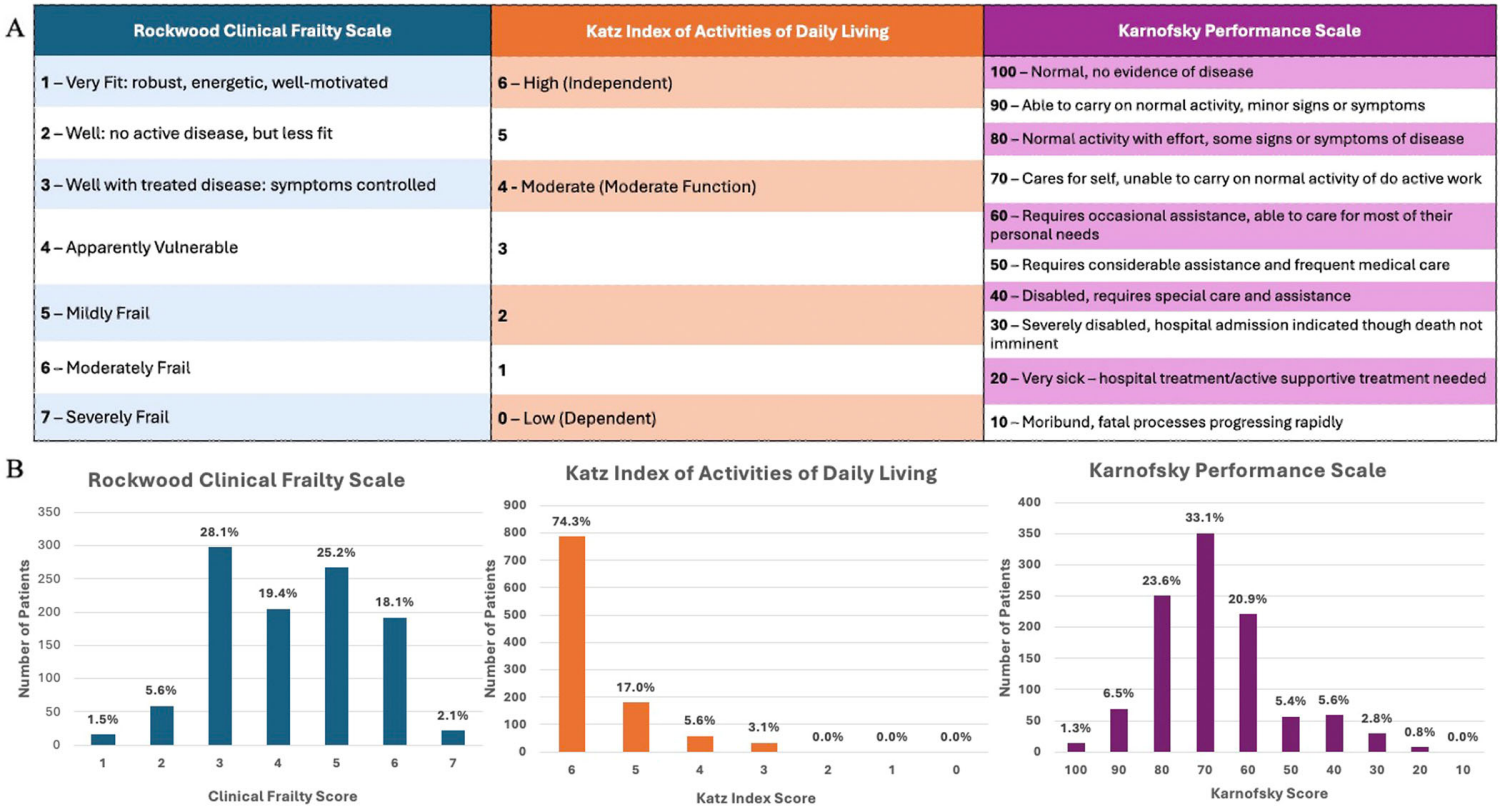
Frailty characteristics of TAVI patients. The above figure demonstrates (A) the scoring scale for each of the three frailty indices and (B) bar charts demonstrating the distribution of patients within each frailty index. [Color figure can be viewed at wileyonlinelibrary.com]

For each patient undergoing TAVI, frailty was assessed using three different clinical frailty indices. Functional baseline and independence in activities of daily living were assessed using both the Karnofsky Performance Index and Katz Index of Activities of Daily Living. Overall frailty was assessed using the Rockwood Clinical Frailty Scale (CFS) (Figure 1A).

All patient data was collected and entered prospectively on a local online database from the point of referral to follow‐up. All individuals who underwent TAVI at our center and who had completed a minimum of 5‐year follow‐up by April 31, 2024, were included in the study. Mortality data were obtained from the NHS Spine (a central NHS framework across the UK) and were available for 100% of patients.

### 2.3 Statistics

Normality of continuous variable distribution was determined using the Shapiro−Wilks test. Continuous variables are displayed as mean ± standard deviation if normally distributed or as median (95% confidence interval [CI]) if non‐normally distributed. Continuous variables were compared using a paired Student's *t*‐test or Wilcoxon signed‐rank test as appropriate. Categorical variables were compared with the use of Fisher's exact test. The restricted mean survival time (RMST) is the average survival time in a pre‐specified period (effectively the area under a Kaplan−Meier curve), providing a more intuitive interpretation than probabilities [14]. Survival for each frailty index is displayed as both 5‐year RMST and survival probability tested using a Cox proportional hazard ratio. Frailty indices were sequestered into three equal‐sized groups representing increasing levels of frailty for this purpose (Katz Index: 6 (1), 5 (2), 1−4 (3); Karnofsky Performance Index: < 50 (1), 50−80 (2), > 80 (3); Rockwood CFS: 1−3 (1), 4−5 (2), 6−9 (3).

Multivariable Cox regression analysis was performed to assess the univariable relationships with mortality. Due to presumed collinearity between each of the frailty indices, three separate multivariable Cox regression models were constructed for each frailty index. Clinical variables were included in the model if they had previously been shown in the scientific literature to be associated with survival and if they met the pre‐specified inclusion significance value (*p* < 0.10) on univariable testing. Receiver operator characteristic curve analysis was performed to compare each of the models for predicting 5‐year survival. Area under the curve (AUC) statistics were compared using the method suggested by DeLong [15]. Data analysis was performed using STATA MP 13.1 for Windows (STATA Corp., College Station, Texas, USA). A *p* value of less than 0.05 was considered statistically significant.

## 3 Results

### 3.1 Baseline Characteristics and Frailty

Between December 2007 and April 2019, 1059 patients underwent TAVI. Baseline characteristics are displayed in Table 1. Median age was 83 years (95% CI: 69−92) and 567 (53.5%) were male. In terms of co‐morbidities, 209 (19.7%) had COPD, 23 (2.2%) had severe liver disease, 251 (23.7%) had peripheral vascular disease, and 133 (12.6%) had suffered either a TIA or CVA. In total, 180 (40.7%) had undergone previous PCI, and 251 (23.7%) had undergone cardiac surgery. Overall, 826 (71%) patients had preserved left ventricular ejection fraction (LVEF) (> 50%), 140 (13.2%) had an LVEF of 30%−49% and 93 (8.8%) had an LVEF of < 30%.

**Table 1:** Baseline characteristics.

Characteristic	TAVI cohort (*n* = 1059)
Age, years (95% CI)	83 (69−92)
Male sex, %	53.5%
Height, cm (95% CI)	169 (162−175)
Weight, kg (95% CI)	75 (64−86)
Creatinine, micromol/L (95% CI)	101 (87−122)
Diabetic, %	20.40%
NYHA class, *n* (%)	
I	8 (0.7%)
II	101 (9.5%)
III	790 (74.6%)
IV	160 (15.1%)
CCS angina, *n* (%)	
1	56 (5.2%)
2	184 (17.4%)
3	90 (8.5%)
4	18 (1.7%)
Extensive calcification of ascending aorta, *n* (%)	209 (13.7%)
Peripheral vascular disease, *n* (%)	251 (23.7%)
History of neurological disease, *n* (%)	
TIA	60 (5.7%)
CVA with full recovery	58 (5.5%)
CVA with residual deficit	15 (1.4%)
COPD, *n* (%)	209 (19.7%)
Severe liver disease, *n* (%)	23 (2.2%)
Previous cardiac intervention	
Balloon aortic valvuloplasty, *n* (%)	117 (11%)
PCI, *n* (%)	180 (17%)
Previous cardiac surgery, *n* (%)	251 (23.7%)
TAVI, *n* (%)	10 (0.9%)

Abbreviations: CCS, Canadian Cardiovascular Society angina classification; COPD, chronic obstructive pulmonary disease; CVA, cerebrovascular accident; NYHA, New York Heart Association Functional Classification; PCI, percutaneous coronary intervention; TAVI, transcatheter aortic valve implantation; TIA, transient ischemic attack.

The frailty characteristics are displayed in Figure 1. A large proportion of patients had a reasonable level of performance with 74.3% identified as having a Katz Index of 6 and 64.5% had a Karnofksy index between 70 and 100, indicating functional independence at baseline. Only 14.5% of patients were deemed to require more than occasional assistance with activities of daily living. Only 45.4% had a Rockwood CFS Score of > 4. In total, 25.2% of patients were felt to be mildly frail, 18.1% moderately frail, and only 2.1% of patients were severely frail at baseline.

### 3.2 Procedural Characteristics

Procedural details are displayed in Table 2. The majority of cases were performed electively (83.9%) using a transfemoral approach (93.0%) across a spectrum of valve platforms. In total, 59 patients (5.6%) underwent valve‐in‐valve for a failing bioprosthesis. The median inpatient stay was 2 days (95% CI: 1−8). Procedural mortality was 0.9%.

**Table 2:** Procedural characteristics.

Procedural characteristic	TAVI cohort (*n* = 1059)
Aortic valve etiology, *n* (%)	
Native, degenerative	1000 (94.4%)
Bioprosthetic, degenerative	59 (5.6%)
Aortic valve characteristics, mean ± SD	
Valve area, cm^2^	0.8 ± 0.2
Mean gradient, mmHg	42.7 ± 16.8
Peak gradient, mmHg	71 ± 25
Left ventricular function (EF%), *n* (%)	
> 50%	826 (78%)
30%−49%	140 (13.2%)
< 30%	93 (8.8%)
General anesthesia, *n* (%)	142 (9.2%)
Procedural urgency, *n* (%)	
Elective	888 (83.9%)
Urgent	165 (15.6%)
Emergency/salvage	6 (0.5%)
Delivery approach, *n* (%)	
Trans‐femoral	985 (93%)
Axillary/subclavian	20 (1.9%)
Transapical	6 (0.5%)
Direct aortic	26 (2.5%)
Valve type, *n* (%)	
Corevalve	469 (44.3%)
Edwards Sapien	178 (16.8%)
Accurate Neo	2 (0.19%)
Lotus	399 (37.7%)
Other	11 (1.0%)

Abbreviation: EF, ejection fraction (%).

### 3.3 Mortality

**Figure 2:**
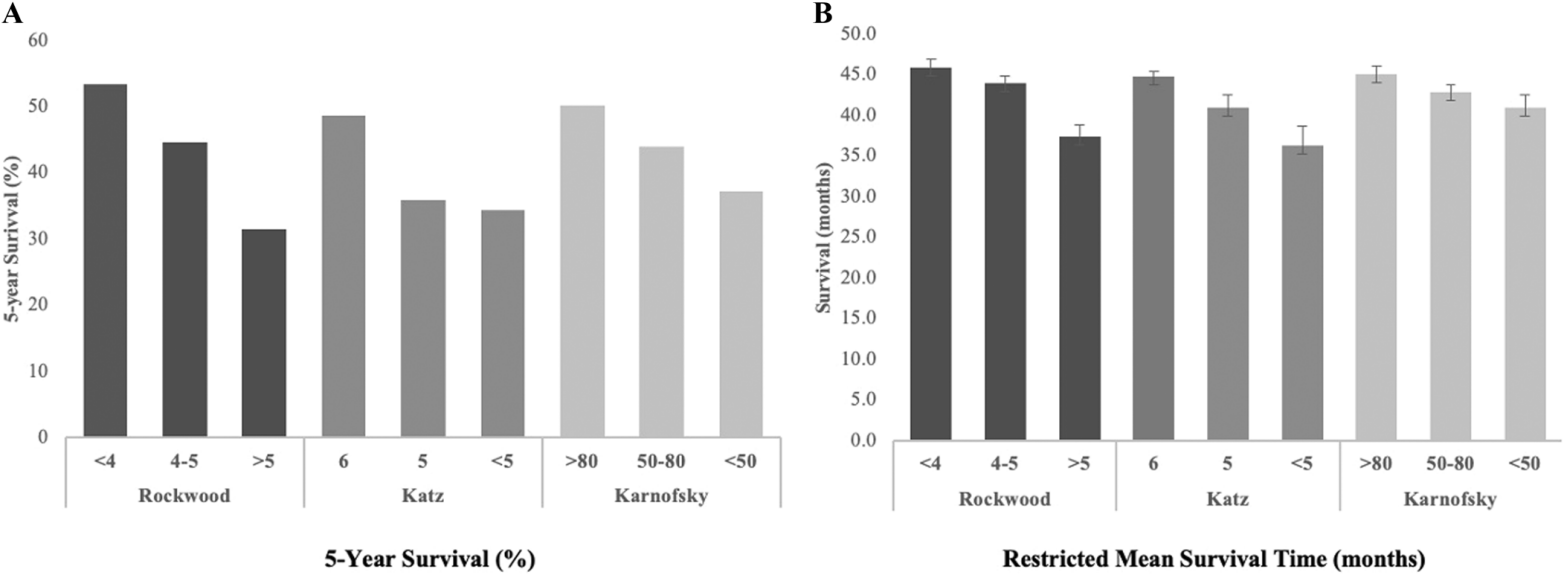
(A) Five‐year survival and (B) 5‐year restricted mean survival time (RMST) according to Rockwood Clinical Frailty Scale, Katz Level of Independence, and Karnofsky performance index. The effect of frailty on survival, as measured through each of the three frailty indices, can be seen.

Median follow‐up for the entire cohort was 1676 days (95% CI: 68−3639). One, three, and five‐year survival was 86.5%, 66.7%, and 44.9%, respectively, and is demonstrated graphically in Figure 2A.

**Figure 3:**
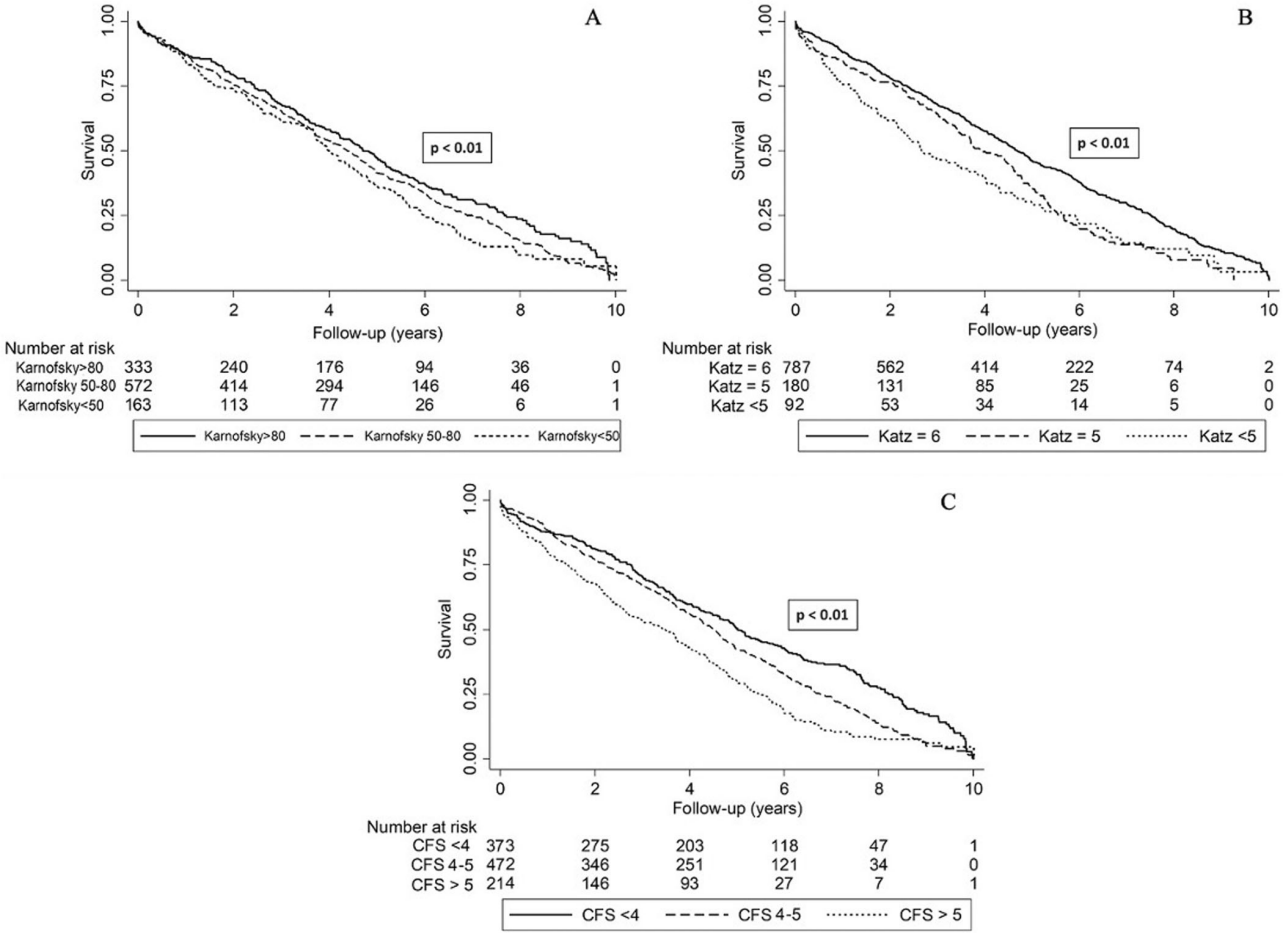
Kaplan−Meier survival curves for (A) Karnofsky performance index, (B) Katz level of independence, (C) Rockwood clinical frailty scale. CFS, Rockwood clinical frailty scale.

All three frailty indices were associated with survival in univariable analysis (Rockwood CFS HR: 1.34, 95% CI: 1.22−1.48, *p* < 0.01; Katz HR: 1.23, 95% CI: 1.11−1.36, *p* < 0.01; Karnofsky HR: 1.21, 95% CI: 1.09−1.34, *p* < 0.01) (Table 3 and Figure 3). Univariable analysis also identified age (HR: 1.02, 95% CI: 1.01−1.03, *p* ≤ 0.01), BMI (HR: 0.98, 95% CI: 0.97−1.00, *p* = 0.01), previous cardiac surgery (HR: 0.87, 95% CI: 0.78−0.97, *p* = 0.02), peripheral vascular disease (HR: 1.28, 95% CI: 1.10−1.50, *p* ≤ 0.01), and preprocedural LV function (HR: 1.19, 95% CI: 1.07−1.31, *p* ≤ 0.01) as clinical characteristics predictive of 5‐year survival (Table 3).

**Table 3:** Univariable and multivariable analysis.

Univariable analysis				Multivariable analysis								
				Katz model			Karnofsky model			Rockwood CFS model		
Variable	HR	95% CI	*p* value	HR	95% CI	*p* value	HR	95% CI	*p* value	HR	95% CI	*p* value
**Katz index**	**1.23**	**1.11–1.36**	**< 0.01**	**1.23**	**1.10−1.37**	**< 0.01**	—	—	—	—	—	—
**Karnofsky performance scale**	**1.21**	**1.09–1.34**	**< 0.01**	**—**	**—**	**—**	**1.25**	**1.11−1.41**	**< 0.01**	—	—	—
**Rockwood clinical frailty scale**	**1.34**	**1.22–1.48**	**< 0.01**	**—**	**—**	**—**	**—**	**—**	—	**1.35**	**1.21−1.50**	**< 0.01**
**Age**	**1.02**	**1.01–1.03**	**< 0.01**	**1.02**	**1.01−10.2**	**< 0.01**	**1.02**	**1.01−1.03**	**< 0.01**	**1.02**	**1.00−1.03**	**0.01**
**Pre‐procedure LV function**	**1.19**	**1.07–1.31**	**< 0.01**	**1.19**	**1.06−1.33**	**< 0.01**	**1.14**	**1.01−1.30**	**0.03**	**1.13**	**1.00−1.26**	**0.046**
**BMI**	**0.98**	**0.97–1.00**	**0.01**	**0.98**	**0.97−1.00**	**0.02**	**0.98**	**0.97−1.00**	**0.02**	0.99	0.98−1.00	0.11
**Previous cardiac surgery**	**0.87**	**0.78–0.97**	**0.01**	**0.88**	**1.13−1.57**	**0.02**	**0.87**	**0.77−0.97**	**0.01**	**0.89**	**0.79−0.99**	**0.04**
**Peripheral vascular disease**	**1.28**	**1.10–1.50**	**< 0.01**	**1.32**	**1.28−1.57**	**0.01**	**1.31**	**1.11−1.54**	**< 0.01**	**1.32**	**1.12−1.56**	**< 0.01**
On dialysis	0.97	0.90–1.05	0.48	**—**	**—**	**—**	**—**	**—**	**—**	**—**	**—**	**—**
Smoking history	1.05	0.92–1.20	0.50	**—**	**—**	**—**	**—**	**—**	**—**	**—**	**—**	**—**
Previous PCI	0.94	0.79–1.13	0.51	**—**	**—**	**—**	**—**	**—**	**—**	**—**	**—**	**—**
Diabetes	1.01	0.94−1.08	0.78	**—**	**—**	**—**	**—**	**—**	**—**	**—**	**—**	**—**
COPD	1.12	0.94 1.31	0.20	**—**	**—**	**—**	**—**	**—**	**—**	**—**	**—**	**—**
Postprocedure AR	1.07	0.98−1.17	0.13	**—**	**—**	**—**	**—**	**—**	**—**	**—**	**—**	

*Note:* Significant variables are displayed in bold with frailty indices displayed at the top of the table. Hazard ratios for the frailty indices reflect increasing levels of frailty utilizing the three sequestered groups for each index as described in the main text above. LV function was ranked as normal (EF > 50%), mild/moderate (EF 30%−50%), or severely impaired (< 30%). Other continuous variables, including age, were incorporated as absolute rather than ranked values. Because of high co‐linearity between the frailty indices, three multivariable models were constructed, incorporating each frailty index separately.

Three multivariable Cox regression models were created, incorporating each frailty index separately. Based upon the pre‐specified inclusion criteria, each of the models also included age, BMI, previous cardiac surgery, peripheral vascular disease, and preprocedural LV function as predictors (Table 3). In each of the three multivariable models, age, pre‐procedure LV function, previous cardiac surgery, and peripheral vascular disease remained predictive of 5‐year survival. BMI was predictive in the Katz (HR 0.98, 95% CI 0.97−1.00, *p* 0.02) and Karnofsky (HR 0.98, 95% CI 0.97−1.00, *p* 0.02) models but did not remain predictive in the Rockwood CFS model (HR 0.99, 95% CI 0.98−1.00, *p* = 0.11) (Table 3).

All three frailty indices were associated with survival in each of the multivariable models (Rockwood CFS HR: 1.35, 95% CI: 1.21−1.50, *p* < 0.01; Katz HR: 1.23, 95% CI: 1.10−1.37, *p* < 0.01; Karnofsky HR: 1.25, 95% CI: 1.11−1.41, *p* < 0.01). ROC curve comparison, however, demonstrated that of the three frailty models, the model which incorporated the Rockwood CFS was the best discriminator of 5‐year survival (Rockwood CFS model: AUC = 0.59 [95% CI: 0.55−0.61], Karnofsky model: AUC = 0.55 [95% CI: 0.52−0.58], Katz model: AUC = 0.55 [95% CI: 0.53−0.58], *χ* ^2^ = 4.43, *p* = 0.04). Pairwise analysis again highlighted the superiority of the Rockwood CFS model (Rockwood CFS model vs. Karnofsky model *χ* ^2^ = 6.67, *p* = 0.01; Rockwood CFS model vs. Katz model *χ* ^2^ = 4.43, *p* = 0.04; Karnofsky model vs. Katz model *χ* ^2^ = 0.03, *p* = 0.86).

## 4 Discussion

This study demonstrates that frailty, as assessed at the time of TAVI, has prognostic implications for long‐term outcome. Using a large cohort followed up over 5 years we have also shown that of the three most commonly used scoring systems, the strongest predictor of survival following TAVI is the Rockwood Clinical Frailty Score.

### 4.1 The Effect of Frailty on Long‐Term Outcomes Following TAVI

Frailty is a disease‐state in itself, and recognition of its presence in older adults is of increasing importance due to the potential for adverse outcomes in these patients following even minor external stressors [16]. Frailty has been shown to be a risk factor for the development of MACE, hospital admissions, falls, and functional decline leading to the requirement for increasing levels of care, even in patients without pre‐existing cardiovascular disease [17, 18]. It is of primary relevance in TAVI patients who are typically more elderly to ensure that patients are likely to obtain both short and long‐term benefit following this procedure.

Surgical risk scores, including STS and EUROSCORE, can be used to assess peri‐procedural risk in TAVI patients. However, they have been found to be suboptimal at predicting outcomes in this patient population [3, 4, 5, 6], and frailty indices rather than cardiac risk scores have been shown to identify functional decline over a 6‐month period following TAVI [19]. Other studies have also demonstrated that adding frailty to these traditional cardiac risk scores can also help to improve prediction of outcomes at 1‐year [20, 21].

The Rockwood CFS has previously been shown to predict outcomes in modest cohort sizes in the short‐term at 1 or 2 years after TAVI [22, 23, 24, 25]. Only one study to date has examined outcomes over a longer time frame, demonstrating frailty as an independent predictor of 5‐year mortality [26]. It is, however, unclear how frailty was assessed in this study, however, and interestingly, in a recent random‐forest machine‐learning algorithm of the same cohort, frailty was excluded from this model [12]. To our knowledge, our study is the first to directly compare different frailty scores over a long‐term follow‐up period. By examining frailty and outcomes over a longer time period, our study has also likely negated the effects of any procedural complications which could potentially impact mortality at both 30 days and 1 year.

### 4.2 The Most Useful Frailty Marker for TAVI Patients

Despite national guidelines encouraging the use of a frailty assessment before TAVI, there is currently no consensus regarding the most appropriate tool to do so, likely as a result of a lack of strong evidence. Two previous studies, including one from our group, have suggested that mobility and/or a Katz index of less than 6 are stronger predictors of poorer outcomes following TAVI [3, 11]. Frailty in these studies was associated with a higher 30‐day mortality but this was not significant after multivariable analysis [11]. These studies, however, included a shorter follow‐up period, with a smaller cohort in one study, which is the likely reason that these more overt markers of frailty and poor health were identified as predictive. As TAVI indications change, the proportion of patients with a Katz score of less than 6 or impaired mobility undergoing TAVI will be less frequent, and less crude measures of frailty will be required to stratify patients.

Furthermore, frailty is a complex disease state which encompasses physical, functional, biochemical, and cognitive domains. In the future, as we understand more about frailty, subjective clinical assessment alone is therefore unlikely to be sufficient, especially given the heterogeneity between different measures and the risk of low‐inter‐rate reliability [11]. Some studies have aimed to try and develop more in‐depth frailty assessment tools, which incorporate multiple different frailty domains. A study by Afilalo et al. in patients undergoing either TAVI or SAVR demonstrated that the Essential Frailty Toolset which included physical, function, and biochemical markers of frailty was the strongest predictor of mortality at 12 months (adjusted odds ratio [OR]: 3.72; 95% CI: 2.54−5.45) compared to numerous frailty scores [27]. Another study looked at a group of patients undergoing TAVI and generated the “Erasmus Frailty Score” which incorporated a mini‐mental state exam, malnutrition universal screening tool (MUST), grip strength, Katz index, and Lawton and Brody Index [28]. They found that the score could predict risk of delirium post‐TAVI and 1‐year mortality [28]. There has also been more recent evidence suggesting that an assessment of frailty combining both functional, metabolic [29] or CT‐based [30] frailty parameters could help to predict 1‐year mortality post‐TAVI insertion. In clinical practice, however, such complex and time‐consuming assessments of frailty are unlikely to always be feasible. This evidence does, however, strengthen the argument for the creation of more objective pre‐procedure predictive models that incorporate a multi‐modal assessment of frailty.

Despite a lack of consensus regarding the most appropriate method to use to assess frailty, current evidence clearly indicates the importance of frailty assessments as part of the decision‐making process in selecting patients for TAVI. Our study, which further demonstrates the importance of frailty as a predictor of both short and longer‐term outcomes in patients following TAVI implantation, serves to strengthen this argument. It also highlights the prognostic ability of the Rockwood CFS compared to the other frailty indices in this patient cohort; however, more work is required to ascertain the best marker of frailty that clinicians should employ in clinical practice given the significant variability of results in previous studies. Until more objective assessments of frailty are designed, it is likely that the use of multiple different frailty indices will still be important to try and make sure that we identify frailty and ensure appropriate patient selection for procedures such as TAVI.

## 5 Limitations

This is one of the first studies to examine long‐term outcomes in TAVI patients in relation to different frailty indices in a large patient cohort. There are, however, some limitations to our study. First, the data has gathered from a single‐center which may affect the external validity of our results. Our population demographics and procedural data, however, reflect similar studies performed in the United Kingdom and worldwide [11, 23]. Second, data has been collected from national NHS Spine data rather than individual local follow‐up data. This is, however, a robust database collecting mortality data, but it does not provide any other outcome measures, and it also does not differentiate between death from all causes and cardiovascular death, which is a limitation. Finally, frailty was assessed by a single TAVI operator rather than a geriatrician or general physician. Although given the recognized presence and importance of frailty in this patient cohort, TAVI operators are often highly experienced at assessing frailty as part of their procedural risk assessment.

## 6 Conclusions

The Rockwood CFS is the strongest predictor of long‐term mortality in patients undergoing TAVI. Our study highlights that frailty assessment is an essential independent tool for predicting long‐term outcomes in these patients.

## Conflicts of Interest

Professor Hildick‐Smith is a proctor for Abbott, Edwards, Medtronic, and Boston Scientific. The other authors declare no conflicts of interest.
